# Case Report: Novel *IL10RB* variant causing very early onset-inflammatory bowel disease

**DOI:** 10.3389/fimmu.2025.1655475

**Published:** 2025-10-06

**Authors:** Yusuf Usman, Christopher P. Ptak, Valeria C. Cohran, Brian E. Nolan, Aisha Ahmed, Aaruni Khanolkar

**Affiliations:** ^1^ Division of Allergy and Immunology, Ann and Robert H. Lurie Children’s Hospital of Chicago, Chicago, IL, United States; ^2^ Biomolecular Nuclear Magnetic Resonance Facility, University of Iowa, Iowa City, IA, United States; ^3^ Department of Molecular Physiology and Biophysics, Carver College of Medicine, University of Iowa, Iowa City, IA, United States; ^4^ Division of Gastroenterology, Ann and Robert H. Lurie Children’s Hospital of Chicago, Chicago, IL, United States; ^5^ Department of Pediatrics, Northwestern University, Chicago, IL, United States; ^6^ Division of Rheumatology, Ann and Robert H. Lurie Children’s Hospital of Chicago, Chicago, IL, United States; ^7^ Department of Pathology, Carver College of Medicine, University of Iowa, Iowa City, IA, United States

**Keywords:** VEO-IBD, IL-10R, phosflow, AlphaFold, HSCT

## Abstract

**Background:**

Very early onset-inflammatory bowel disease (VEO-IBD) can arise from monogenic defects affecting immune regulation. We report a male child with VEO-IBD caused by a homozygous, loss-of-function *IL10RB* variant (c.562T>G; p.C188G) that has not been previously reported for this disorder.

**Case Presentation:**

A male infant of Hispanic descent was admitted to our hospital at the age of 8 months due to intractable colitis, perianal fistulas and growth faltering. Endoscopy at nine months of life revealed pancolitis and gastritis. Despite multiple courses of steroids and use of sulfasalazine, their disease remained active. Standard biologic therapies (infliximab and adalimumab) were trialed in the second year of life without improvement. Given the very early onset and severe phenotype, functional testing by phosflow to evaluate the IL-10 signaling pathway demonstrated the absence of STAT3 phosphorylation in response to IL-10 and follow up genetic testing identified a novel homozygous *IL10RB* missense variant (c.562T>G; p.C188G). Subsequent protein structure analysis using AlphaFold corroborated this loss-of-function phenotype. The patient’s condition was partially controlled with anakinra (IL-1 receptor antagonist) as a bridge therapy. At the age of 3 years, the patient underwent an allogeneic hematopoietic stem cell transplant (HSCT) from an unrelated umbilical cord blood donor; however, they experienced engraftment failure, likely due to persistent hyperinflammation and the choice of cord blood for HSCT. The patient continues to have active disease requiring on-going medical management and supportive care.

**Conclusion:**

We report a novel, loss-of-function *IL10RB* variant causing VEO-IBD, thus expanding the genotypic spectrum of this condition. This case highlights the diagnostic and therapeutic challenges of IL-10R deficiency–related VEO-IBD. It also underscores the importance of early recognition of monogenic causes of IBD, use of interim immunomodulatory therapies, and the need for optimal timing and donor selection for HSCT.

## Introduction

**Figure 1:**
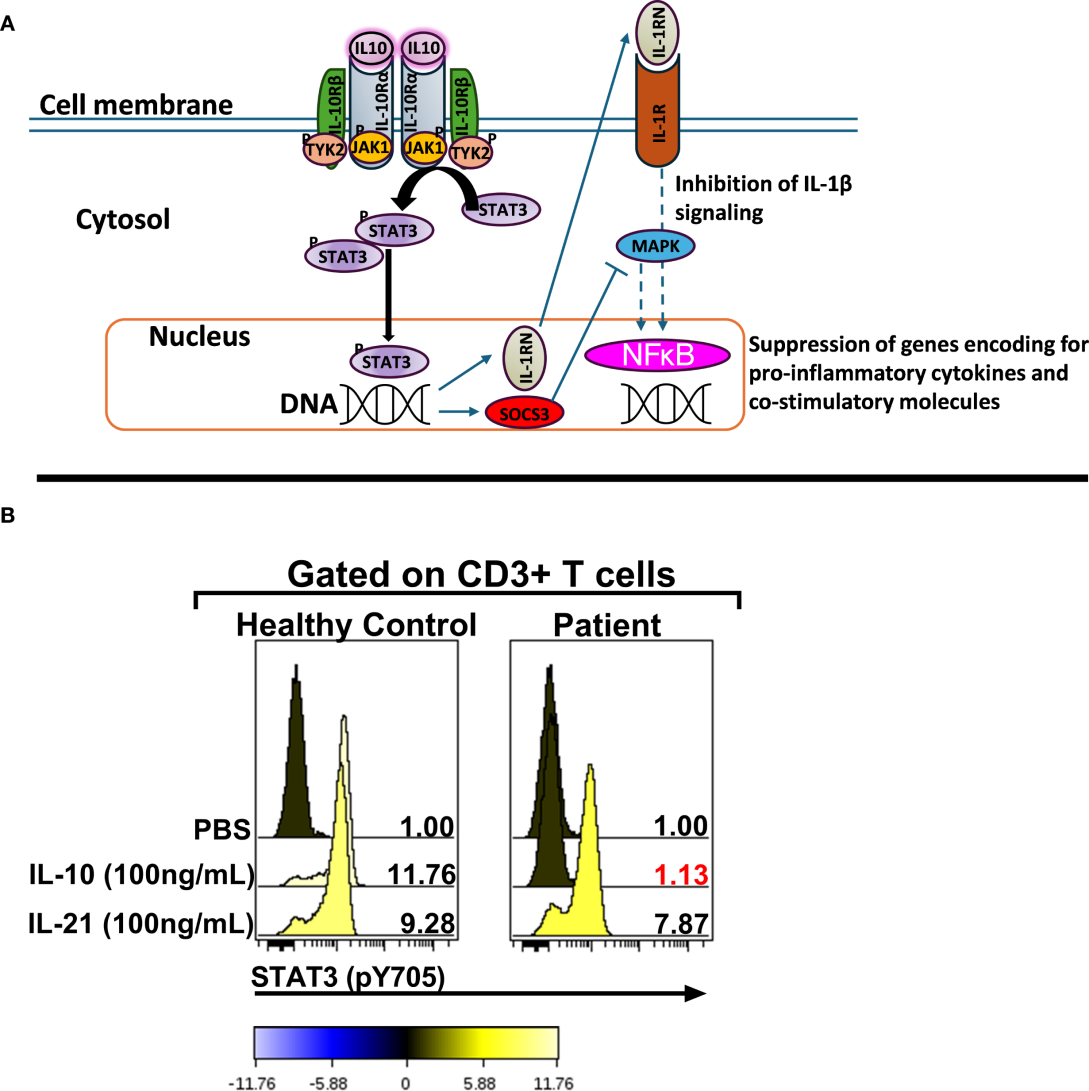
The *IL10RB* (c.562T>G; p. C188G) variant abrogates IL-10 signaling. **(A)** Schematic representation of the IL-10 signaling pathway [adapted from Schulke, S et al] (6). **(B)** Assessment of IL-10 signaling in bulk CD3+ T cells. The numerical values in each stacked histogram plot denote the fold change in the median fluorescence intensity (MFI) of the phospho-STAT3 signal following treatment with the signaling input compared to PBS treatment which is normalized to one. The change in the phosphorylation status is additionally depicted by the color of each histogram based on the colorimetric scale placed below the plot. STAT3: Signal Transducer and Activator of Transcription 3; SOCS3: Suppressor of Cytokine Signaling 3; IL-1RN: IL-1 receptor antagonist; JAK1: Janus Kinase 1; Tyk2: Tyrosine kinase 2; MAPK: Mitogen-activated protein kinase; IL-10R: IL-10 Receptor.

VEO-IBD defined as IBD presenting before 6 years of age, can be caused by an underlying primary immunodeficiency (1, 2). One such cause is interleukin-10 signaling pathway deficiency, due to variants in either the IL10 gene (encoding the anti-inflammatory cytokine IL-10) or in its receptor subunits IL10RA and IL10RB (3, 4). IL-10 normally binds a tetrameric receptor composed of two IL-10Rα and two IL-10Rβ subunits, triggering a signaling cascade through JAK1/Tyk2 and STAT3 that suppresses pro-inflammatory cytokine release (**Figure 1A**) (5, 6). Defects in IL-10 or IL-10R disrupt this regulatory pathway, resulting in uncontrolled intestinal inflammation from infancy (4). Over 60 cases of IL-10/IL-10R deficient VEO-IBD have been documented to date (4). Patients present in infancy with refractory diarrhea, pancolitis, and perianal fistulizing disease; growth failure is common (1, 2, 7). Some patients also develop extraintestinal manifestations such as arthritis or folliculitis (1, 2). Conventional IBD therapies often fail to induce remission in IL-10R deficiency, although temporary partial responses to steroids or anti-tumor necrosis factor agents have been documented (7). The only curative treatment is HSCT, which can restore IL-10 signaling; and outcomes are best when performed early in life, before irreversible complications arising from chronic inflammation ensue (8). In this report, we describe a Hispanic male child with VEO-IBD due to a novel *IL10RB* variant, whose disease course illustrates the characteristic phenotype and challenges in management of IL-10R deficiency–associated IBD.

## Methods

### Phosflow staining and analysis

Phosflow staining and analysis was performed as previously described (9–11). Whole blood samples were treated with PBS or recombinant human (rh) IL-10 (BD Biosciences, San Jose, CA) or rhIL-21 (Biolegend, San Diego, CA) and stained with monoclonal Abs targeting CD45 (clone HI30, ThermoFisher Scientific, Waltham, MA); cytoplasmic CD3 (clone UCHT1) and the phospho-epitope of STAT3 (pY705)(clone 4/P-STAT3) (both from BD Biosciences, San Jose, CA). The lymphocytes were identified based on CD45 vs sideways light scatter (SSC) properties and hierarchical gating was utilized to identify CD3+ lymphocytes. A total of 50,000 events were acquired using a FACS Canto II flow-cytometer (BD Biosciences, Franklin Lakes, NJ) and data were analyzed using Cytobank software (version 5.0) (Beckman Coulter, Indianapolis, IN).

### Case description

The patient is a male child born to parents who are first cousins, with no family history of inflammatory bowel disease or immune disorders. The patient was born full-term with normal birth weight and initially appeared healthy. At around 6–7 months of life, the patient was referred to the pediatric gastroenterology service due to persistent diarrhea (often with blood), hepatitis, along with poor weight gain and severe perianal dermatitis (**Table 1**). By 8 months of life, a perianal abscess developed that required surgical drainage. The patient was again referred to pediatric gastroenterology at that time for evaluation of intractable diarrhea and failure to thrive.

**Table 1:** Summary of clinical milestones.

Age	Clinical Events and Interventions
6–8m	Onset of chronic diarrhea, poor weight gain; perianal abscess formation. First pediatric GI evaluation at 8 months of age.
9.5m	Endoscopy confirmed gastritis and severe pancolitis.
8–18m	Recurrent colitis flares; multiple steroid courses. Sulfasalazine started with minimal effect. Perianal fistula managed with seton placement.
2.5y	Anti-TNF biologics (infliximab, then adalimumab) trialed with no response.
3y	Phosflow analysis reveals complete absence of IL-10–induced STAT3 phosphorylation. Subsequent WES identifies a novel, homozygous, loss-of-function *IL10RB* variant (c.562T>G; p.C188G)→ Unrelated, 5/8 HLA allele matched cord blood HSCT performed that failed to successfully engraft, followed by HLH-like hyperinflammation
3y- present (12y)	Initiation of anakinra (IL-1Rα blocker) and canakinumab (IL-1β blocker) for inflammation control. Partial clinical improvement noted with IL-1 pathway inhibition agents and prolonged course of steroids. Diverting ileostomy performed at the age of 7y. Following bowel resection, a re-diversion of the ileostomy was performed two years later. Ongoing moderate colitis.

On examination at 8 months of life, the infant was determined to be underweight. Initial laboratory tests showed mild anemia and hypoalbuminemia. Inflammatory markers were only modestly elevated despite obvious clinical inflammation. A diagnostic endoscopy was performed at 9.5 months of age that revealed diffuse gastritis, and colonoscopy showed severe pan-colitis with deep ulcerations throughout the colon. Histopathology of the biopsies demonstrated chronic active colitis consistent with IBD. No pathogens were identified on special stains or cultures. A diagnosis of infantile-onset IBD was made (initially categorized as IBD-unclassified due to overlapping Crohn/colitis features).

The patient was started on total parenteral nutrition (TPN) as well as corticosteroids to control the acute inflammation. Several courses of high-dose steroids were required during infancy to manage flares of colitis. Once the patient’s condition stabilized, they were transitioned to sulfasalazine (5-aminosalicylic acid) as a maintenance therapy at around 1 year of age. In the United States, sulfasalazine is routinely used as front-line therapy for VEO-IBD to limit steroid exposure even in the absence of extra-intestinal manifestations. However, disease activity remained high: the patient continued to have frequent diarrhea, poor growth, and persistent perianal fistula drainage. Over the course of the next twelve months, the patient experienced multiple colitis flares requiring steroid treatment despite being on sulfasalazine. A repeat endoscopy performed around 2.5y of life confirmed ongoing pancolitis with new inflammation observed in the terminal ileum, indicating the disease had extended. During this time, surgical intervention was required for perianal disease including abscess formation.

Given the refractory nature of the patient’s IBD, biologic therapy was attempted. The patient was started on infliximab (anti-TNF monoclonal antibody), initially at standard dosing (5 mg/kg). Due to lack of a clinical response, the dose was quickly escalated to 10 mg/kg and the dosing interval shortened. Despite these adjustments, the patient’s gastrointestinal symptoms did not improve; in fact, the condition worsened, and infliximab was discontinued after two infusions. The patient was then switched to adalimumab (subcutaneous anti-TNF). Similarly, adalimumab failed to induce any remission of their colitis, and it was discontinued after about 6 weeks. At this stage, all conventional IBD therapies had been exhausted without success.

**Figure 2:**
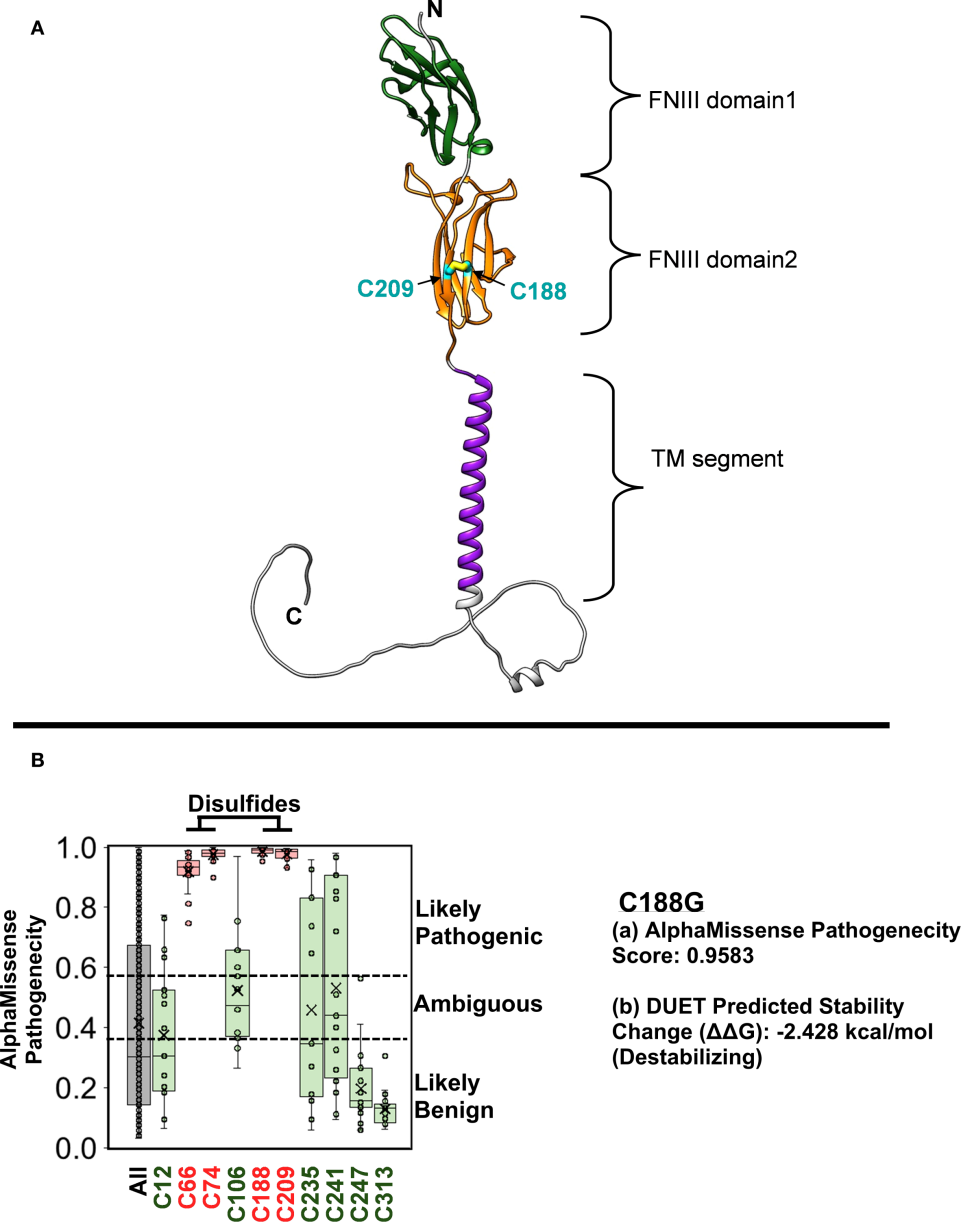
The structure of the IL-10Rβ protein implicates C188G in disease pathogenicity. **(A)** The signal peptide-removed IL-10Rβ AlphaFold structure (AF-Q08334-F1) is composed of multiple domain regions. The two FNIII domains are extracellular and bind directly to the IL-10 ligand. These are followed by a lipid bilayer-embedded transmembrane segment and an unstructured cytosolic region. C188, located on the 2^nd^ FNIII domain, participates in a disulfide bond with C209. **(B)** AlphaMissense Pathogenicity scores predict the likelihood that an amino acid change will be disease-causing. The box plots show scores for the 19 natural amino acids changes at each cysteine position in IL-10Rβ. Only the 4 cysteines involved disulfides, which include C188, were predicted to be fully conserved with any change leading to a likely pathogenic protein. A box plot for all amino acid changes for all positions (ALL) was included to show the range of scores throughout the protein. Impairment of disulfide bond formation in the C188G IL-10Rβ protein is predicted to be highly destabilizing and expected to alter its structure and function. The final 17 amino acids were remodeled for illustration purposes.

From the time of initial presentation, an underlying genetic/immunological cause of early, severe IBD was suspected. Genetic testing was initiated at presentation (in the neonatal period) because of a family history of consanguinity and infant deaths. Initially, targeted genotyping for *BIRC4* was ordered given that 4% of pediatric patients that present with IBD harbor an X-linked Inhibitor of Apoptosis (XIAP) deficiency (12). This testing revealed a wild-type *BIRC4* gene. Whole exome sequencing (WES) was recommended at the age of 1 year of life (2013) and completed in the 3y of life (2015) after receiving parental consent and guarantee of insurance coverage. An important point to consider is that in 2013 WES was not widely available as a clinical test in the US and receiving results of WES testing took far longer that it does now. The patient was evaluated by the immunology team starting at 8.5 months of life. Routine immunologic workup was unremarkable (**Table 2**). Serum cytokines levels were not obtained at this stage because at and around the time of the transplant (2015), clinically-orderable serum cytokine testing was very limited in the United States. Furthermore, even now there’s considerable debate surrounding the validity of the results associated with serum cytokine testing owing to the lack of standardization in the testing methodologies utilized by clinical laboratories that offer these tests (13). However, given the clinical presentation there was a strong suspicion for an IL-10 pathway defect, therefore the integrity of the IL-10 signaling pathway was interrogated by phosflow (**Figure 1B**) (9–11). This analysis revealed a complete abrogation of STAT3 phosphorylation following treatment of the patient’s whole blood sample with recombinant human IL-10 (rhIL-10) (**Figure 1B**). Treatment of the patient’s whole blood sample with rhIL-21, that also engages STAT3, revealed a normal phospho-STAT3 signature suggesting a defect upstream of STAT3 within the IL-10 signaling axis (**Figure 1B**). The result of this functional test was corroborated by subsequent WES that confirmed a novel, homozygous *IL10RB* c.562T>G (p.C188G) variant. This variant results in a cysteine-to-glycine substitution at amino acid 188 in the IL-10Rβ protein, which is predicted to disrupt a conserved disulfide bond in the receptor’s extracellular domain. Both experimentally determined and modeled IL-10Rβ protein structures support this conclusion (14, 15). The C188 residue forms a disulfide bond with C209, which is critical for maintaining proper folding of the second extracellular FNIII domain (**Figure 2A**) (16). Substitution with glycine removes this bond, likely destabilizing the extracellular domain’s conformation and further preventing the formation of a functional IL-10R complex. This was corroborated by AlphaMissense, which predicted the C188G variant to be *highly pathogenic* (score: 0.9583, near the maximum of 1), and by DUET, which predicted significant protein destabilization (ΔΔG: **–**2.428 kcal/mol) (**Figure 2B**) (17, 18). These structure-based predictions align with the clinical phenotype and functional assay confirming absent IL-10R signaling in this patient. Collectively, these analyses conclusively established IL-10Rβ deficiency as the underlying cause for the VEO-IBD in this patient.

**Table 2:** Immunology laboratory values for the patient.

Parameter	Initial Value (Day 39 of life)	Latest Value (Day 4605 of life; Age in years: 12)
IgM	80.8 mg/dL (RR: 19–83 mg/dL)	54.6 mg/dL (RR: 49–230 mg/dL)
IgG	1050 mg/dL (RR: 241–870 mg/dL)	1460 mg/dL (RR: 584–1509 mg/dL)
IgA	89.0 mg/dL (RR: 1.3–53 mg/dL)	738 mg/dL (RR: 45–234 mg/dL)
IgE	73.2 KU/L (RR: <5.2 KU/L)	356 KU/L (RR: <192 KU/L)
CD3^+^ T cells	1488/mm³ (RR: 1375–7129 cells/mm³)	1203/mm³ (RR: 1051–3031 cells/mm³)
CD4^+^ T cells	1221/mm³ (RR: 1169–5623 cells/mm³)	482/mm³ (RR: 548–1720 cells/mm³)
CD8^+^ T cells	267/mm^3^ (RR: 267–1860 cells/mm³)	689/mm^3^ (RR: 332–1307 cells/mm³)
B cells	176/mm^3^ (RR: 104–1448 cells/mm³)	153/mm^3^ (RR: 203–1139 cells/mm³)
NK cells	123/mm^3^ (RR: 60–434 cells/mm³)	24/mm^3^ (RR: 138–1027 cells/mm³)

RR, Reference Range.

With this definitive diagnosis, curative therapy via HSCT was considered. In the interim, the care team implemented alternative immune-modulating treatment to control the ongoing inflammation. At 3 years of life the patient proceeded to receive an HSCT. The stem cell donor was unrelated, with a 5/8 HLA allele match, selected due to the absence of a matched sibling or fully matched unrelated adult donor. Immediately prior to initiating the conditioning regimen, the patient had worsening diarrhea requiring total parenteral nutrition [Day (-) 26 and Day (-)13], with elevated serum CRP and ferritin, and decreased serum albumin. The team, however, proceeded with HSCT due to severity of the patient’s disease and inadequate disease control with previous therapy. The patient underwent a reduced intensity conditioning regimen [fludarabine x 6 days, busulfan x 2 days, anti-thymocyte globulin (ATG) x 4 days, and thiotepa 5mg/kg] followed by the unrelated, umbilical cord blood transplant that unfortunately failed to successfully engraft. The transplant complication was primary graft failure with the recovery and persistence of autologous hematopoietic cells rather than donor-immune cell mediated rejection. Chimerism testing revealed that donor cell engraftment was below the limit of detection of the assay (<2% donor cells on Day 17 and Day 24 post-transplant). There was no clinical evidence of GVHD (Graft vs Host Disease). There was laboratory evidence of post-transplant adenovirus exposure (adenovirus PCR was positive in stool and nasopharynx on D24 post-transplant). However, the patient did not display clinically-overt evidence of adenoviral infection and there is no documentation of disseminated adenoviral disease or another systemic infection following the transplant. Hence, it is likely that on-going and persistent inflammation (related to the patient’s IBD) around the time of transplant and the fact that the patient received a mismatched, unrelated donor cord-blood transplant might have contributed to the graft failure (19–22).

The post-transplant period was marked by a severe hyperinflammatory syndrome with features of hemophagocytic lymphohistiocytosis (HLH), driven by the patient’s native hyperactive immune cells. High-dose corticosteroids and additional immunosuppressants were required during this period to control this HLH-like episode. The patient’s CBC counts initially rose but then declined as the graft was rejected; by two months post-transplant, the patient’s cells had fully reverted to autologous hematopoiesis harboring the original IL-10R deficiency. This was confirmed by re-testing the IL-10 signaling potential in the patient cells by phosflow (data not shown). After the failed HSCT, the patient was started on anakinra, (a recombinant IL-1 receptor antagonist) as an off-label therapy. Following the initiation of daily anakinra injections, systemic inflammation and stool frequency improved partially, but due to ongoing breakthrough symptoms and intestinal disease, was switched to canakinumab at a dose of 4 mg/kg every 4 weeks. He has done best on this agent, but continues to have episodic flares of disease, and has required a prolonged course of steroids as well as supplemental parenteral nutrition. As depicted in **Figure 1A**, IL-10 signaling can induce the expression of IL-1R antagonist (IL-1RN) which can then inhibit IL-1 signaling and negatively regulate the expression of genes encoding for proinflammatory cytokines (6). This was the primary rationale for treating with patient with anakinra and canakinumab. The patient also underwent a diverting ileostomy at the age of 7y. Following bowel resection, a re-diversion of the ileostomy was performed two years later.

The patient is currently 12y old and remains under close clinical observation and is being maintained on monthly canakinumab and supportive care, which help to partially control disease symptoms. The patient continues to have moderate gastrointestinal symptoms (approximately 3–5 loose stools per day) with occasional perianal drainage. His current length, weight, and BMI parameters Z-scores are -2.6, -1.2, 0.45 respectively. On therapy, our patient’s serum albumin has improved into the normal range and C-reactive protein is low; however, fecal calprotectin and erythrocyte sedimentation rate are intermittently elevated, reflecting intestinal inflammation. The medical team and family have discussed the possibility of a second HSCT in the future, potentially with a different donor source (such as a well-matched adult donor or a haploidentical relative), in hopes of ultimately curing their disease.

This data demonstrates that the patient did not have lymphopenia or hypogammaglobulinemia at baseline and is consistent with prior reports that IL-10R–deficient patients often have normal immune profiles aside from aberrations in the IL-10 signaling pathway (3). The elevated IgA and IgE levels on follow-up likely reflect chronic immune stimulation from ongoing intestinal inflammation, a phenomenon noted in some IL-10R–deficient patients (1, 2, 23).

## Discussion

Our patient’s presentation and course align with the classic phenotype of IL-10R deficiency. The patient developed severe colitis during the first year of life, with diffuse colonic inflammation and multiple perianal fistulas, consistent with prior reports of IL-10 pathway variants (3, 4). Like many other patients with IL-10R deficiency-associated VEO-IBD, our patient failed to respond to conventional IBD therapies. The genetic diagnosis confirmed the need for definitive treatment via HSCT.

To date, over sixty cases of *IL10* or *IL10R* variants causing VEO-IBD have been reported worldwide (4). Most patients have homozygous variants in *IL10RA* or *IL10RB*, inherited in an autosomal recessive manner (often with a family history of consanguinity). The *IL10RB* variant identified in our patient (c.562T>G, p.C188G) is novel and it likely abolishes IL-10Rβ function by disrupting a crucial cysteine residue in the receptor’s extracellular domain. Phosflow analysis of the IL-10 signaling pathway and protein structure analysis utilizing the AlphaFold Database strongly supported this conclusion. One of the limitations of the study is that we did not directly assess the surface expression of IL-10RB by flow-cytometry. However, we firmly believe that the functional flow testing (phosflow) that we performed more than adequately identifies that defect in the IL-10 signaling pathway and it also clearly demonstrates that the defect lies upstream of STAT3. Notably, IL-10Rβ is a shared subunit for IL-22 and type III interferons, yet the clinical presentation of IL10RB deficiency is mainly intestinal (14). This was evident in our patient, whose illness was limited to enterocolitis with no significant infections.

The patient’s disease course demonstrated the limited efficacy of standard IBD therapies in the context of IL-10R deficiency. Corticosteroids provided a slight improvement in symptoms, but they became steroid-dependent due to a lack of better long-term options. Sulfasalazine (oral 5-ASA) had little to no impact on controlling their colitis. Biologic agents targeting TNF (infliximab and adalimumab) also failed to induce remission, which is consistent with reports that IL-10R–deficient IBD generally does not respond to anti-TNF therapy (24). These treatment failures, combined with the early age of onset, strongly pointed to an underlying immunoregulatory defect rather than typical polygenic IBD.

HSCT is currently the only curative therapy for IL-10R deficiency, and early transplantation is associated with the best outcomes (8). In the case of our patient the decision was made to use an available partially-matched, unrelated umbilical cord blood unit for transplantation, since no matched donor existed. Unfortunately, as described, the HSCT was unsuccessful. Our patient’s failed transplant underscores the challenges when HSCT is delayed. The failure to achieve engraftment was likely multifactorial including the patient’s highly activated immune state at the time of transplant. Despite pre-transplant conditioning, their immune system mounted an overwhelming inflammatory response (similar to HLH) that created a hostile environment for the donor stem cells. Similar hyperinflammatory transplant complications have been reported in other IL-10R deficient patients who underwent HSCT later in life (25). Additionally, cord blood transplants are known to have slower engraftment kinetics and a higher risk of graft failure in older or obese pediatric patients, due to limited cell dose and naive immune cells (22). This highlights the importance of controlling disease activity before transplant and carefully selecting the donor source. A second transplant for our patient will likely involve a well-matched adult donor, aggressive pre-transplant immunosuppression and maximal control of inflammation to improve the chances of success.

The patient’s response to canakinumab adds to emerging evidence that IL-1 blockade can serve as a useful bridge therapy in IL-10R deficiency (26). A prior case documented that anakinra induced clinical improvement and even resolution of an extraintestinal manifestation (IgA nephropathy) in a child with IL10RA deficiency (27). Similarly, our patient’s inflammatory markers improved on anakinra and subsequent canakinumab and both agents were well tolerated. Other medical therapies have been tried in cases of refractory VEO-IBD with mixed success (e.g. ustekinumab), and JAK inhibitors (like ruxolitinib) are under consideration to control hyperinflammation before the next transplant attempt in our patient (28). Because the IL-10 “brake” is not functional in this case, other pro-inflammatory cytokines that signal via JAKs (such as IL-6) can have pronounced inflammatory effects. Furthermore, our phosflow data showed that STAT3-mediated signaling was preserved for IL-21 which indicates that the downstream JAK-STAT mechanism is still intact(**Figure 1B**). Additionally, IL-6, a potent pro-inflammatory cytokine, also signals through JAK and STAT3 (29). Therefore, treatment with JAK inhibition is warranted even in the face of absent IL-10 signaling to dampen hyperinflammation as a bridge therapy prior to HSCT.

Nutritional rehabilitation has also been emphasized to improve our patient’s growth and resilience; they receive a high-calorie oral and enteral diet with parenteral nutrition supplementation to address their nutritional needs. Surveillance colonoscopies and imaging are performed as needed to monitor disease activity and complications (such as fistula tracts). At this juncture, a multidisciplinary team (including pediatric gastroenterologists, immunologists, and transplant specialists) is deliberating a second HSCT attempt. The consensus is that any future transplant should aim for identifying a fully-matched, unrelated, adult donor in addition to maximal control of inflammation.

In summary, this case highlights that clinicians should consider a monogenic cause in any infant with severe, refractory IBD (especially with perianal disease). Furthermore, our report expands the *IL10RB* variant spectrum for VEO-IBD and contributes to understanding genotype-phenotype correlations. Sharing such case experiences will help improve future diagnosis and treatment of this rare disease.

## Data Availability

The original contributions presented in the study are included in the article/supplementary material. Further inquiries can be directed to the corresponding authors.
